# A179 UNDERSTANDING THE NEED FOR A ZINC DIET TO TREAT ZINC DEFICIENCY IN PATIENTS WITH CELIAC DISEASE

**DOI:** 10.1093/jcag/gwad061.179

**Published:** 2024-02-14

**Authors:** S Tandon, K Graham, L Russell, J Morgan, D Armstrong, M Pinto-Sanchez

**Affiliations:** Medical Sciences, McMaster University Faculty of Health Sciences, Hamilton, ON, Canada; Medical Sciences, McMaster University Faculty of Health Sciences, Hamilton, ON, Canada; Farncombe Family Digestive Health Research Institute, Hamilton, ON, Canada; Farncombe Family Digestive Health Research Institute, Hamilton, ON, Canada; Medicine, McMaster University Faculty of Health Sciences, Hamilton, ON, Canada; Medical Sciences, McMaster University Faculty of Health Sciences, Hamilton, ON, Canada

## Abstract

**Background:**

Nutritional deficiencies in celiac disease (CeD) are caused by delayed intestinal healing and a nutrient-lacking gluten-free diet (GFD). At our specialized adult celiac clinic, zinc (Zn) was the most common deficiency present in patients and is clinically treated through Zn supplementation. However, Zn supplements in excess may cause inhibition of other minerals, and gastrointestinal side effects. Alternatively, a Zn diet may be a sustainable option to treat zinc deficiency, however patient preference regarding this treatment method is unknown.

**Aims:**

To understand patient preference of zinc supplementation compared to a zinc diet to treat Zn deficiency in CeD. We also aim to determine the tolerability and effectiveness of Zn supplements to reduce gastrointestinal and extraintestinal symptoms in the CeD population.

**Methods:**

This observational prospective pragmatic study was conducted from March 2022-September 2023. Eligible participants had bloodwork indicating plasma Zn levels ampersand:003C 9.4 μ/mol . All participants were prescribed 25 mg per day of Zn gluconate supplements throughout the study. Patients were provided a RedCap link where they completed questionnaires at Visit 1 and at their next clinic appointment in 3-6 months (Visit 2). These surveys included; Celiac Symptom Index (CSI) Questionnaire 2) Extraintestinal Symptoms Visual Analogue Scale (ES-VAS) and 3) Patient Perception Survey (PPS). Continuous data are expressed as Mean (SD), and categorical data is reported as frequencies (%). The Wilcoxon test was used to assess changes in symptoms.

**Results:**

A total of 42 participants completed the Visit 1 survey, of which 17 participants had their follow up clinic appointment (Visit 2). Out of these 17 participants, 15 were female (88.2%) and the mean age was 33.7 years (+/- 10.6). The mean CSI score at Visit 1 was 35.3 (+/- 10.3) and 33.7 (+/- 13.0) at Visit 2, indicating moderate disease symptoms (p=0.552). The most common extraintestinal symptoms was anxiety (ES-VAS: 5.7), foggy mind (ES-VAS: 4.1), fatigue (ES-VAS: 4.1) and depression (ES-VAS: 3.5). There were no significant changes in extraintestinal symptoms between clinic visits. Out of 42 participants, 15 (35.7%) prefer to treat their Zn deficiency through diet, 26 (59.5%) through supplement, and 1 (2.4%) of participants had no preference. Additionally, 35 (85.4%) participants indicated that they were “likely” to comply to a Zn-diet treatment.

**Conclusions:**

CeD patients did not experience improvement in gastrointestinal or extraintestinal symptoms on Zn supplementation. However, a portion of CeD patients prefer to treat and are likely to comply with a Zn diet to treat their deficiency. Based on this knowledge, further studies should investigate the efficacy of a Zn diet compared to supplements CeD patients.

**Funding Agencies:**

CIHR

